# A novel approach to generate a recombinant toxoid vaccine against *Clostridium difficile*

**DOI:** 10.1099/mic.0.066712-0

**Published:** 2013-07

**Authors:** Robert G. K. Donald, Mike Flint, Narender Kalyan, Erik Johnson, Susan E. Witko, Cheryl Kotash, Ping Zhao, Shakuntala Megati, Irina Yurgelonis, Phillip Kwok Lee, Yury V. Matsuka, Elena Severina, Anne Deatly, Mini Sidhu, Kathrin U. Jansen, Nigel P. Minton, Annaliesa S. Anderson

**Affiliations:** 1 Pfizer Vaccine Research, Pearl River, NY 10654, USA; 2 Centers for Disease Control, 1600 Clifton Rd, Atlanta, GA 30333, USA; 3 Clostridia Research Group, NIHR Biomedical Research Unit in GI Disease, University Park, University of Nottingham, Nottingham NG7 2RD, UK

## Abstract

The *Clostridium difficile* toxins A and B are primarily responsible for symptoms of *C. difficile* associated disease and are prime targets for vaccine development. We describe a plasmid-based system for the production of genetically modified toxins in a non-sporulating strain of *C. difficile* that lacks the toxin genes *tcdA* and *tcdB*. TcdA and TcdB mutations targeting established glucosyltransferase cytotoxicity determinants were introduced into recombinant plasmids and episomally expressed toxin mutants purified from *C. difficile* transformants. TcdA and TcdB mutants lacking glucosyltransferase and autoproteolytic processing activities were ~10 000-fold less toxic to cultured human IMR-90 cells than corresponding recombinant or native toxins. However, both mutants retained residual cytotoxicity that could be prevented by preincubating the antigens with specific antibodies or by formalin treatment. Such non-toxic formalin-treated mutant antigens were immunogenic and protective in a hamster model of infection. The remaining toxicity of untreated TcdA and TcdB mutant antigens was associated with cellular swelling, a phenotype consistent with pore-induced membrane leakage. TcdB substitution mutations previously shown to block vesicular pore formation and toxin translocation substantially reduced residual toxicity. We discuss the implications of these results for the development of a *C. difficile* toxoid vaccine.

## Introduction

*Clostridium difficile* is a spore-forming Gram-positive bacillus producing exotoxins A and B (TcdA and TcdB) which are pathogenic to humans. *C. difficile* is the primary cause of antibiotic related infectious diarrhoea in elderly hospitalized patients in developed countries (Simor *et al.*, 2002). Symptoms of *C. difficile* associated disease (CDAD) range from diarrhoea to severe colitis, toxic megacolon, sepsis and death. Over recent years, increases in disease incidence, severity and recurrence are largely due to the emergence of hypervirulent strains associated with epidemic hospital outbreaks combined with an increase in resistance to commonly used antibiotics (reviewed by Rupnik *et al.*, 2009). The antibiotics metronidazole and vancomycin are commonly used to treat the most serious cases, but relapses are common. Fidaxomicin, an antibiotic licensed in 2011 for treatment of CDAD, has the ability to reduce rates of recurrence compared with vancomycin with similar overall efficacy (Louie *et al.*, 2011). Moreover, the drug is expensive and cost-effectiveness remains to be established (Whitman & Czosnowski, 2012). By circumventing the need for antibiotic intervention altogether, a prophylactic vaccine capable of neutralizing the *C. difficile* A and B exotoxins would offer a much needed alternative approach for preventing this devastating disease.

Toxins A and B are very large proteins of 308 kDa and 270 kDa that are structurally related, sharing homologous functional domains that mediate intracellular uptake and delivery of a cytotoxic glucosyltransferase (GT) (reviewed by Jank & Aktories, 2008). Binding of toxin C-terminal domains to cell-surface receptors leads to endocytosis and fusion with endosomal vesicles. The acidic pH of the endosomal lumen is thought to trigger a conformational change in each toxin that induces pore formation, translocation and cytosolic exposure of the GT domain. Autoproteolytic cleavage mediated by the cysteine protease domain and its cofactor inositol 6-phosphate releases the GT enzyme to the cytosol. The resulting glucosylation and irreversible inactivation of Rho family GTPases causes disruption of the actin cytoskeleton leading to apoptosis and cell death. Although the toxins differ individually in their potency and effects in *in vivo* models, studies in hamsters suggest that they both contribute to disease in natural infections (Kuehne *et al.*, 2010; Lyerly *et al.*, 1985). Furthermore, vaccination with both toxin A and toxin B – but not with either alone – conferred protection in hamster models of infection (Libby *et al.*, 1982).

Recognition of the ability of the humoral immune response to control CDAD stems from the successful use of passive immunotherapy with pooled human immunoglobulin containing anti-toxin A and B antibodies to treat severe CDAD (Salcedo *et al.*, 1997). Furthermore, reduction in recurrence of CDAD was achieved in a Phase I clinical trial with A and B toxin monoclonal antibodies in combination with standard antibiotic therapy (Lowy *et al.*, 2010). In addition, in a small study with three patients with chronic relapsing CDAD, an investigational vaccine using formalin-inactivated A and B toxoid antigens prevented CDAD recurrence (Sougioultzis *et al.*, 2005). Collectively, these observations provide validation for, and encourage further development of *C. difficile* toxin A- and B-based vaccines to prevent CDAD.

The large-scale manufacturing of *C. difficile* toxins for vaccine development presents safety challenges, including exposure to toxins and decontaminating facilities of heat-resistant spores. Fortunately, recent molecular biological advances offer potential solutions. The ClosTron mutagenesis procedure for targeted stable insertional inactivation of *C. difficile* genes has permitted the construction of strains unable to form spores (Heap *et al.*, 2007; Underwood *et al.*, 2009). Another technological development is a versatile *Escherichia coli–Clostridium* plasmid shuttle vector system that permits episomal expression of recombinant antigens (Heap *et al.*, 2009). This modular platform offers a range of selectable markers and *E. coli–Clostridium* replicons that can be empirically tailored for optimal outcome. We have used both genetic systems to explore the feasibility of safely producing genetically inactivated toxins in their native cellular environment, one that is naturally adapted for the production and secretion of these large antigens (Govind & Dupuy, 2012). With this goal in mind, site-directed mutations were introduced to neutralize previously defined cytotoxicity determinants including catalytic amino acid residues responsible for GT activity, autoproteolytic release of this domain and recognition of Rho GTPase substrates (Busch *et al.*, 1998; Egerer *et al.*, 2007; Jank *et al.*, 2007b). Re-evaluation of the impact of these mutations on the cytotoxicity of A and B toxins revealed a level of residual toxicity that appears to be unrelated to GT activity. The discovery that much of this toxicity can be eliminated by mutagenic targeting of residues shown to facilitate pore formation (Genisyuerek *et al.*, 2011) confirms the need to address multiple cytotoxic mechanisms for genetic or chemical inactivation of these toxin antigens.

## Methods

### 

#### Strains, plasmids and conjugal transfer.

*C. difficile* strains were grown anaerobically in Brain Heart Infusion (BHI) media or on agar (OXOID) supplemented with 0.5 % yeast extract and 0.1 % cysteine (BHIS). An anaerobic workstation (Whitley model MG1000) operating with a standard gas mixture (10 % H_2_, 10 % CO_2_ and 80 % N_2_) was used for all experiments. *C. difficile* strains 630 and VPI 10463 were obtained from ATCC (numbers BAA-1382, 43255). A previously described erythromycin-sensitive variant of strain 630 known as 630Δ*erm* was used as host for erythromycin-resistant plasmids (Hussain *et al.*, 2005). For 4–8 l fermentations, bacteria were grown in modified HySoy Medium (20 g HySoy, 5 g yeast extract, 0.9 g KH_2_PO_4_, 5 g NaHCO_3,_ 10 g glucose, 1 g thioglycolate per litre).

Synthetic *tcdA* and *tcdB* genes were designed bearing double allelic substitutions in key GT catalytic site residues (D285A/D287A for toxin A; D286A/D288A for toxin B). The recombinant genes were based on strain 630 toxin genome sequences (Sebaihia *et al.*, 2006) and were further modified to remove internal restriction endonuclease recognition (RE) sites useful for subcloning into *C. difficile* plasmid vectors (Heap *et al.*, 2009). Following synthesis (Blue Heron Biosciences), full-length *tcdA* and *tcdB* genes were subcloned as respective 8.1 kb and 7.1 kb *Nde*I–*Bgl*II fragments into vector pMTL84123 using standard molecular biology techniques. Plasmid pMTL84123 was constructed as described (Heap *et al.*, 2009). *E. coli* strain Stbl2 (Invitrogen) was used as host for stable maintenance of recombinant plasmids prior to conjugative transfer to *C. difficile*. TcdA and TcdB plasmid transformants were selected on Miller’s LB agar with chloramphenicol (30 µg ml^−1^) at 30 °C. *C. difficile* promoter fragments were PCR-amplified from strain 630 and subcloned into vector pMTL82254 using 5′ and 3′ flanking *Not*I and *Nde*I RE sites. Mutations to further modify or revert the double mutant A or B toxins were introduced by site-directed mutagenesis of internal 2.5 kb *Nde*I–*Hin*dIII or 3.3 kb *Nde*I–*Eco*NI fragment subclones, respectively, using a QuikChange kit (Stratagene). Modified fragments were exchanged after restriction digestion, fragment purification and ligation and mutations confirmed by DNA sequencing using an ABI3730 instrument (Life Technologies). Oligonucleotides used for PCR cloning or mutagenesis are summarized in Table S1, available in *Microbiology* Online. ClosTron insertional mutants of VPI 10463 and GC-8126 strain *spo0*A genes were constructed using pMTL007E-C2 plasmids as described (Heap *et al.*, 2007; Heap *et al.*, 2010). Retargeted sequences were designed (www.clostron.com) and directed mutagenesis plasmids ordered from DNA 2.0.

Conjugal transfer of plasmids from *E. coli* to *C. difficile* was performed as described (Heap *et al.*, 2009) with minor modification for optimal maintenance of recombinant 14 kb and 13 kb *tcdA* and *tcdB* plasmids. Intermediate *E. coli* host strain CA434 harbouring the Tra+ Mob+ R702 conjugative plasmid was used as donor strain. Plasmid transformants were grown in Miller’s LB with chloramphenicol (30 µg ml^−1^) at 30 °C to mid-exponential phase. Bacterial cultures (2 ml) were harvested by centrifugation (5000 ***g***) and washed in PBS to remove antibiotic. Cell pellets were transferred to an anaerobic growth chamber and suspended in 0.2 ml late-exponential-phase *C. difficile* recipient freshly grown in BHIS media. The mixture was spotted on BHIS agar and after 16 h of growth at 37 °C, cell patches were scraped into 0.5 ml PBS and 0.1 ml plated on BHIS agar supplemented with 15 µg ml^−1^ thiamphenicol (to select exconjugants) and d-cycloserine/cefoxitin (to kill *E. coli* donor cells). Transformants appearing 16–24 h later were purified by restreaking onto a BHIS agar (plus supplements) and subsequent cultures tested for expression of genetically modified toxins. Serial passaging of transformants in BHIS media in the absence of selection revealed a 3 % and 1 % plasmid loss per generation for TcdA and TcdB plasmids, respectively (Fig. S1). As a result, thiamphenicol was routinely added to growth media for the 4–8 l fermentations used for the purification of recombinant toxins. To verify plasmid integrity, plasmid DNA minipreps were prepared from 2 ml BHIS cultures using a modified Qiagen kit procedure in which cells were pretreated with lysozyme. *C. difficile* miniprep DNA was used as a template for sequencing overlapping PCR fragments to verify clone integrity. Alternatively, plasmid DNA of higher purity was prepared from *E. coli* Stbl2 transformants and directly sequenced.

#### Preparation of bacterial lysates.

*C. difficile* plasmid transformants were grown anaerobically at 37 °C in BHIS media in 2 ml Eppendorf tubes (for routine analysis) or as 50 ml cultures in 125 ml vent-capped flasks (for time-course experiments). Bacteria samples (2 ml) were harvested by centrifugation (9000 ***g***, 30 s) and culture supernatants retained and concentrated 10-fold by spin-column filtration (Amicon-ultra30 kDa). Cell pellets were drained, subjected to one freeze–thaw cycle [−80 °C, room temperature (RT)] and resuspended in 1 ml lysis buffer (10 mM Tris/HCl pH 7.5, 1 mM EDTA, 15 % glycerol). The suspension was sonicated with 1×20s burst of a microtip (Branson). The lysate was centrifuged at 4 °C (18 000 ***g***, 10 min) and the resulting supernatant concentrated 10-fold as before. Samples of supernatant and lysate were combined with sample buffer and heat treated (10 min, 80 °C) before loading onto 3–8 % Tris/acetate SDS-PAGE gels (Invitrogen). Gels were stained with Coomassie dye (SimplyBlue Safe stain, Invitrogen) to visualize individual proteins.

#### Chloramphenicol acetyltransferase assay.

To quantify chloramphenicol acetyltransferase activity, 5 µg crude lysate total protein was incubated with 3.5 µl [^14^C]chloramphenicol (50 mCi mmol^−1^, PerkinElmer) and 5 µl 40 mM acetyl-coenzyme A (Sigma) in 50 µl Tris–EDTA pH 7.5 buffer for 30 min at RT. The acetylated products and unmodified reactants were separated from the aqueous solution by organic extraction with 300 µl ethyl acetate. The organic phase was removed, lyophilized and resuspended in 10 µl ethyl acetate before spotting on a silica thin-layer chromatography plate (Baker). Mono- and di-acetylated chloramphenicol was separated from unacetylated forms by ascending chromatography in chloroform/methanol solvent (95 : 5). The per cent conversion of [^14^C]chloramphenicol to acetyl-[^14^C]chloramphenicol was measured by PhosphorImager analysis of the dried TLC plate (ImageQuant software, GE Healthcare).

#### Toxin antibody ELISA.

A sandwich ELISA was established to quantify toxin or toxin mutants in crude extracts or lysates. High binding Maxisorb (Thermo) ELISA plates were coated with 3 µg ml^−1^ anti-toxin A or anti-toxin B rabbit polyclonal sera (Meridian Life Sciences) in PBST buffer (PBS+ 0.05 % Tween-20) for 1 h at 37 °C. Plates were blocked in 10 % Superblock buffer (Thermo), washed and incubated with test samples or serially diluted TcdA or TcdB standard (0.2–200 ng ml^−1^ for A; 2–2000 ng ml^−1^ for B). After washing, bound antigen was detected following stepwise incubations with 1 µg ml^−1^ A or B toxin-specific mAbs (Meridian Life Sciences) and horseradish peroxidase (HRP)-conjugated anti-mouse antibody (Jackson). A TMB substrate kit (Thermo) was used for colorimetric determination. Concentrations of antigens in test samples were interpolated from standard curves using the four-parameter equation (GraphPad Prism).

#### Cell culture and cytotoxicity assays.

Human fetal lung IMR-90 cells (ATCC) were cultivated under standard conditions in MEM culture medium (Gibco) [supplemented with heat-inactivated 10 % FBS, 0.01 % gentamicin and 1 % non-essential amino acids (NEAA)]. Cells were dispensed to 96-well tissue culture plates at a density of 1×10^4^ cells per well. Experimental toxin or toxoid samples were serially diluted in MEM assay medium (1 % heat-inactivated FBS, 0.01 % gentamicin and 1 % NEAA) and added to IMR-90 cells 24 h after plating. The assay plate was incubated at 37 °C for 72 h. In some cases, plates were visually inspected for cytopathic effects (characterized by cell rounding), following incubation for 24 h. After 72 h, cell viability was determined using the bioluminescent CellTiter-Glo reagent (Promega), measuring ATP release upon cell lysis. Cytotoxicity was expressed as an EC_50_, equivalent to the amount of toxin or toxoid causing 50 % reduction in luminescence. Dose–response curves were fitted with the four-parameter equation in GraphPad Prism.

The xCelligence System (Roche) was used for the continuous monitoring of toxin-treated IMR-90 cells growing on gold microelectrode-coated microplate wells. A reduction in detected impedence reflects loss of cellular contact resulting from detachment, death or change in shape. Cells were seeded at a density of 1×10^4^ cells per well in culture medium and allowed to grow for 24 h with impedence recorded every 15 min. After 24 h, media was replaced with test samples serially diluted in MEM assay medium and cells monitored for an additional 72 h at 37 °C.

#### Toxin neutralization assay.

Threefold serial dilutions of each hamster serum sample were prepared and mixed with fixed concentrations of wt TcdA or TcdB (eight times the EC_50_ value for each toxin) and incubated at 37 °C for 90 min in a humidified incubator (5 % CO_2_). Subsequently, the toxin-antiserum mixtures were added to IMR-90 cell monolayers on 96-well plates, which were incubated for an additional 72 h. Subsequently, the cytotoxicity within each well was measured using the CellTiter-Glo reagent as described. The RLU values were plotted against the dilution of the test serum samples to generate a four-parameter logistic regression response fit curve. The neutralization titres were expressed as the sample dilution which exhibited a 50 % reduction in cytotoxicity.

#### *In vitro* autocatalytic cleavage assay.

Cleavage assays were performed in 0.1M Tris-HCL pH 7.5, at a final volume of 20 µl with 1 µg wt or mutant toxin. The reactions were initiated by the addition of inositol hexakisphosphate (InsP6; Sigma, at final concentration of 10 µM for toxin A and 100 µM for toxin B) and 2 mM DTT. Following incubation for 90 min at RT, the reaction was stopped by the addition of an equal volume of 1× Laemmli Buffer (Biorad) and heated at 95 °C. Protein samples were analysed by SDS-PAGE and detected by silver stain (Bio-Rad).

#### Glucosylation assay.

*In vitro glucosylation assay*: Each reaction contained 30 µM UDP-^14^C-glucose (~3.3 µCi; American Radiolabelled Chemicals), 50 mM HEPES, pH 7.2, 100 mM KCl, 4 mM MgCl_2_, 2 mM MnCl_2_, 1 mM DTT, 0.1 µg µl^−1^ BSA and 10 µg RhoA GTPase. TcdA or TcdB proteins were added and reactions incubated at 30 °C for 2 hr. The protein samples were denatured at 70 °C, for 10 min in loading buffer and separated on 4–12 % SDS-PAGE (Invitrogen). Gels were fixed in 40 % methanol, 10 % acetic acid, 3 % glycerol for 30–60 min and dried. After exposure to a storage phosphor screen overnight, images were captured on a PhosphorImager (GE Healthcare). Alternatively, glucosylation reactions were spotted on GFA filters (GE) pre-soaked in 10 % TCA. Filters were dried briefly and washed extensively with 10 % TCA followed by 100 % ethanol to remove unincorporated label. Dried filters were placed into vials containing 5 ml scintillation fluid and counted. 

*In vivo glucosylation assay:* IMR-90 cells grown in 6-well plates were treated with toxin B (500 pg ml^−1^) or TcdB mutant (100 ng ml^−1^) for 24, 48, 72 and 96 h. The morphology of treated IMR-90 cells was monitored daily by light microscopy. Following incubation, media was removed. Cells were transferred by scraping the monolayer into lysis buffer [50 mM Tris/HCl, pH 7.2, 1 % (w/v) Triton X-100, 500 mM NaCl, 10 mM MgCl_2_] and incubated for 20 min on ice. After centrifugation (18 000 ***g***, 20 min, 4 °C), supernatants were analysed by SDS-PAGE (4–12 % acrylamide; Invitrogen) and Western blotting to monitor glucosylation of the cellular RacGTPase substrate. Rac1 GTPase was quantified with mAb clone 23A8 (Upstate) and the unglucosylated enzyme with mAb clone 102 (BD), respectively. An actin antibody was used as reference control and a colorimetric Western Breeze kit (Invitrogen) was used for detection.

#### Purification of wild-type toxins, recombinant toxins or toxin mutants and formalin treatment of isolated proteins.

Wild-type and mutant toxins A and B were isolated from 8 l cultures grown under anaerobic conditions using a combination of anion-exchange chromatography on Q-Sepharose and hydrophobic chromatography on Phenyl-Sepharose columns (GE Healthcare). An additional chromatographic step on hydroxyapatite was introduced for further purification of recombinant TcdA proteins from cell lysates (as opposed to culture supernatants). Formalin-treated toxoids were prepared by incubating purified proteins with a mixture of formaldehyde and glycine (40 mM each) for 24 h at RT.

#### Preparation of *C. difficile* spores.

Cultures of *C. difficile* strain 630 were grown on BHI agar plates anaerobically at 37 °C for 7 days, harvested into 10 ml PBS using disposable loops, and the spores were processed as described (Sambol *et al.*, 2002). Spores were enumerated by plating 0.1 ml of 10-fold serial dilutions on BHI agar plates supplemented with 1 % taurocholic acid, incubating anaerobically at 37 °C for 30–40 h, then counting the colonies on each plate and multiplying by the dilution factor.

#### Animals.

All animal care and procedures conformed to Institutional Animal Care and Use Committee guidelines. The facilities were fully accredited by the American Association for Accreditation of Laboratory Animal Care. Hamsters were obtained from Charles River Laboratories and housed one per cage. Prior to antibiotic administration, hamsters were housed in autoclaved cages containing autoclaved bedding, food and water to prevent possible environmental contamination.

#### Immunogenicity and challenge study.

On weeks 0, 4, 8 and 12, anaesthetized animals (*n* = 5) were immunized with a mixture of toxoids (10 µg toxoid A and 10 µg toxoid B in buffer containing 500 µg ml^−1^ AlPO_4_) by the intramuscular route (0.05 ml per hamster). One group of hamsters (*n* = 5) was injected with saline to serve as an infection control. Prior to each immunization and prior to the challenge (week 16), hamsters were anaesthetized with 105–150 µg kg^−1^ dexdomitor, administered intraperitoneally, and approximately 0.2 ml blood was collected from the tarsal vein of each hamster. A final blood collection was taken by cardiac puncture following scheduled or unscheduled euthanasia. At 4 weeks after the last immunization (week 16), hamsters were given clindamycin antibiotic (30 mg kg^−1^ in 1.0 ml PBS, by orogastric route) to disrupt the normal intestinal flora. Five days later, *C. difficile* 630 spores (5×10^3^ c.f.u. in 1.0 ml DMEM (Gibco) per hamster, given by the orogastric route) were administered.

To determine protection from CDAD, the hamsters were monitored using a clinical scoring system to track disease severity. Hamsters were monitored as frequently as 6–7 times in an 18 h window and the following parameters were scored using a weighted system: abnormal activity (scored 0, 2 or 4); hunched posture (scored 0 or 2); stimulus response (scored 0, 1 or 2); skin tent (scored 0, 1 or 2); sunken eyes (scored 0, 1 or 2); loose faeces (scored 0 or 1); wet tail (scored 0, 2, 4 or 7); diarrhoea (scored 0 or 3); tender abdomen (scored 0 or 3); rough or oily coat (scored 0, 1 or 2); and body temperature drop (scored 0 or 3 if animals dropped >2 °F body temperature in less than 3 h). Rather than using death as an end point, animals determined to have severe CDAD, as marked by total severity score ≥15, were euthanized. In addition, any animal found to be moribund was euthanized immediately.

## Results

### Identification of a *C. difficile* host strain for production of mutant toxins

We sought to express mutant non-toxic forms of the *C. difficile* TcdA and TcdB toxins to evaluate their utility as vaccine antigens. Rather than using a heterologous system to produce these large proteins, we used *C. difficile* as the expression host. To prevent recombination events between chromosomal and plasmid toxin genes and to simplify the purification process we selected a non-toxigenic *C. difficile* strain for antigen production. Naturally occurring non-toxigenic *C. difficile* strains lacking the pathogenicity locus (PaLoc) have previously been identified by multiplex PCR assay (Braun *et al.*, 1996). Using the same assay we identified an additional *C. difficile* strain, GC-8126, lacking the PaLoc. This strain could acquire shuttle vector plasmids with erythromycin or thiamphenicol selectable markers at an acceptable frequency by conjugal transfer (~10^−8^ per *E. coli* donor). A PCR assay (Gonçalves *et al.*, 2004) was used to prove that this strain also lacked the genes for a secondary toxin known as binary toxin that encodes an actin specific ADP-ribosyltransferase. To eliminate the capacity of the strain to form infectious heat–resistant spores, we inactivated the *spo0A* gene by ClosTron-mediated site-directed insertional mutagenesis. SpoA is the master transcriptional regulator controlling sporulation initiation in spore-forming Gram-positive bacteria (reviewed by Stephenson & Lewis, 2005). Inactivation of the gene in *C. difficile* strain 630Δ*erm* by ClosTron-mediated insertion completely eliminated spore-forming ability (Underwood *et al.*, 2009). We constructed an analogous asporogenic *spo0A* mutant in *C. difficile* GC-8126, and confirmed that it was unable to form spores (data not shown).

### Evaluation of promoters for episomal gene expression

**Fig. 1.**
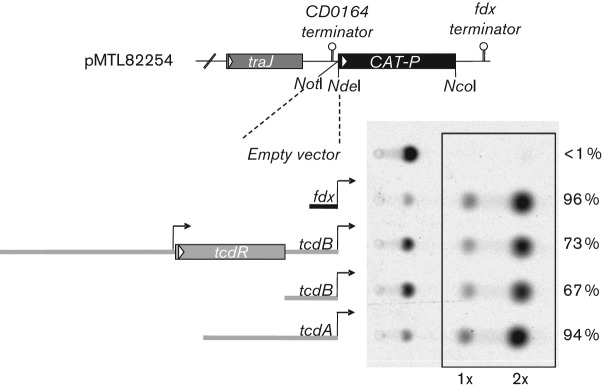
Evaluation of promoters for plasmid-based gene expression. *C. difficile* toxin and *C. sporogenes fdx* gene promoter fragments were subcloned in front of the CAT reporter in vector pMTL82254 (Heap *et al.*, 2009) and plasmid constructs transferred by conjugation to *C. difficile* strain 630Δ*Erm*. Transcriptional terminators, *traJ* conjugal transfer gene and flanking *Not*I and *Nde*I cloning sites are also illustrated. Crude total protein (5 µg) from lysates of stationary phase cultures were incubated with ^14^C-chloramphenicol and acetyl-CoA for 30 min at room temperature. CAT activity was determined by PhosphorImager analysis of radiolabelled components resolved by TLC. Mono- and bi-acetylated products are highlighted and the per cent conversion from un-acetylated substrate is indicated for each construct.

The native promoters that mediate expression of *C. difficile tcdA* and *tcdB* genes are subject to multiple levels of transcriptional regulation that increase toxin production under conditions of nutrient limitation while suppressing yields during exponential growth (Antunes *et al.*, 2012; Dineen *et al.*, 2007). To maximize expression of recombinant toxoids in standard culture media, we sought a promoter from which gene expression was relatively insensitive to regulation by nutrients or phase of growth. We evaluated a number of potential promoters including the *Clostridium sporogenes* feredoxin (*fdx*) promoter, a component of the modular *Clostridium* shuttle vector system (Heap *et al.*, 2009), as a possible alternative to *tcdA* and *tcdB* gene promoters. Plasmids were constructed in which candidate, *C. difficile tcdA* or *C. difficile tcdB* gene promoters were fused to the chloramphenicol acetyltransferase (CAT) reporter gene and comparatively evaluated for CAT expression in stationary phase cultures of *C. difficile* strain 630Δ*erm*. In the experiment shown in Fig. 1, the *fdx* promoter construct expressed higher levels of CAT activity than either of two *tcdB* promoter constructs, one of which included the upstream *tcdR* regulatory gene. The *fdx-CAT* and *tcdA*-CAT fusions expressed reporter activity at comparable levels under these conditions. Based on these results, the *C. sporogenes fdx* gene promoter was selected for further investigation.

### Expression and activity of TcdA and TcdB mutants

**Fig. 2.**
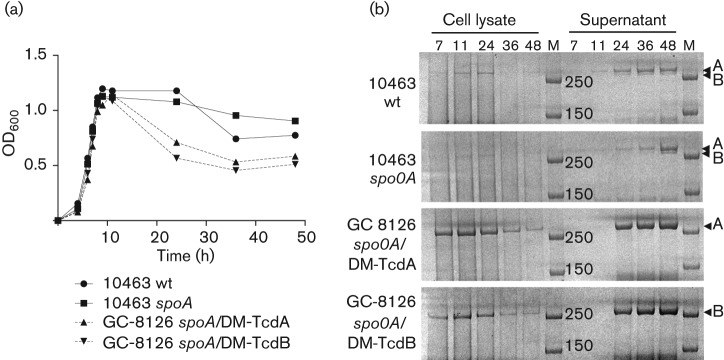
TcdA and TcdB expression in wt and recombinant toxin production strains. (a) Bacterial growth curves of strains expressing native toxins (VPI 10463, solid lines) and recombinant double mutant (DM) antigens (GC-8126, dotted lines). (b) TcdA and TcdB expression in cell pellets and in culture supernatants at 7, 11, 24, 36 and 48 h time points. Crude cell lysates and culture supernatants were separated by SDS-PAGE and high molecular mass toxins/toxoids visualized with Coomasie stain. Samples were concentrated 10-fold by membrane filtration before loading the gel. Arrowheads indicate location of full-length TcdA and TcdB proteins. M, molecular mass markers (250 and 150 kDa).

Plasmid vector pMTL84123 (Heap *et al.*, 2009), comprised of a low copy number *E. coli* replicon and conjugation transfer sequence, the *C. difficile* pCD6 replicon, the CAT selectable marker and the *C. sporogenes fdx* promoter, was chosen for expression of recombinant TcdA and TcdB antigens. Synthetic *tcdA* (8.1 kb) and *tcdB* (7.1 kb) genes with double substitution mutations known to inactivate cytotoxic GT activity – D285A/D287A for toxin A and D286A/D288A for toxin B (Busch *et al.*, 1998; Teichert *et al.*, 2006) – were inserted separately behind the *fdx* promoter. Plasmids were introduced by conjugation from *E. coli* into *C. difficile* GC-8126-*spoA*178a : : CT. Expression of the TcdA and B double mutants (DM-TcdA and DM-TcdB) in individual transformants was compared to wt *C. difficile* VPI 10463, a well-established producer of native A and B toxins (Fang *et al.*, 2009). In the time-course experiment shown in Fig. 2, robust intracellular expression of recombinant DM-TcdA and DM-TcdB was observed throughout exponential and early stationary phases of growth, followed by an accumulation of the proteins in the culture supernatant after 24 h that was accompanied by partial cellular lysis (Fig. 2a). Substantially lower levels of native A and B toxins were detected for strain VPI 10463. Yields of native A and B toxins were found to be similar in an isogenic VPI 10463 strain *spoA* ClosTron insertional mutant, indicating that inactivation of the *spoA* gene had no influence on production of native toxins under these conditions. The concentration of DM-TcdA and DM-TcdB in the culture supernatant at the 48 h time point was determined in both cases by sandwich ELISA to be 75±20 µg ml^−1^ (*n* = 2). This represents a significant increase in protein output compared to the level of native toxin A previously reported for *C. difficile* VPI 10463 (Fang *et al.*, 2009).

**Fig. 3.**
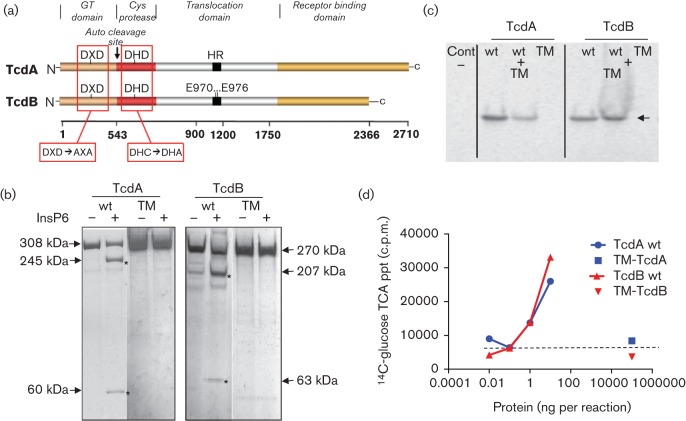
Structural features and GT activities of TM toxins. (a) Schematic highlighting mutations targeting GT catalytic and auto-proteolytic processing activities, modified from illustration in Jank *et al.* (2007a). Also indicated is the position of pore-inducing glutamate residues E970 and E976 within the TcdB hydrophobic region (HR). (b) InsP6-induced auto-proteolytic cleavage of purified wt TcdA/B and TM-TcdA/B toxins. Purified proteins (~1 µg) were incubated in the presence or absence of InsP6 at RT overnight. The cleavage products were separated by SDS-PAGE and stained with silver. Asterisks mark the molecular sizes of proteolytic cleavage products. (c) GT activity of native toxins and TM toxins. wt TcdA/B (1 ng) or TM-TcdA/B (100 µg) were incubated with RhoA GTPase in the presence of UDP-^14^C-glucose for 2 h at 30 °C. The activity of 100 µg TM-TcdA/B in the presence of 1 ng wt TcdA/B (wt+TM) was also assessed as control. The reaction mixture was resolved on SDS-PAGE and radioactive bands were visualized by PhosphorImager. Arrow indicates the 28 kDa ^14^C-labelled Rho GTPase protein band. (d) Concentration dependent toxin GT activity (at >0.1 ng ml^−1^) and lack of detectable activity (at 100 µg) for TM-antigens. After 2 h at 30 °C, reaction products were TCA precipitated and counted. Dotted line indicates the level of background ^14^C-glucose counts recovered from control reactions lacking TcdA or TcdB protein.

To further reduce the potential for cellular cytotoxicity, the catalytic cysteine residues responsible for autocatalytic processing of TcdA (C700) and TcdB (C698) proteins were targeted by site-directed mutagenesis (Egerer *et al.*, 2007; Fig. 3a). The C700A and C698A alleles were introduced into the respective DM-TcdA and DM-TcdB plasmids to create triple mutant (TM) constructs, which were transferred to *C. difficile* strain GC-8126-*spoA*178a : : CT for expression of recombinant antigens. Mutant toxins were purified and incubated in the presence of InsP6, the natural cytosolic cofactor shown to facilitate autoproteolytic cleavage (Reineke *et al.*, 2007). Unlike the native A and B toxins that yielded expected cleavage products, both TM-TcdA and TM-TcdB failed to exhibit any evidence of auto-cleavage, even after prolonged incubation (Fig. 3b). GT activity of the TM toxins was assessed by measuring the *in vitro* glucosylation of RhoA GTPase with UDP ^14^C-glucose substrate. Radioactive incorporation of ^14^C-glucose into RhoA GTPase was monitored by SDS-PAGE and by TCA precipitation (Fig. 3c, d). Wild-type TcdA and TcdB toxins showed efficient transfer of ^14^C-glucose to RhoA GTPase at a concentration of 1 ng each per reaction. In contrast, neither of the TM toxins incorporated detectable levels of radiolabelled glucose in either gel-based or precipitation assays even at 100 µg per reaction. Spiking of 100 µg of either TM toxin with 1 ng of the respective native toxin in the reaction restored GT activity, showing that the observed lack of activity was not due to non-specific inhibition by potential contaminants in the recombinant protein preparations. In similar experiments, no GT activity was detected when either Rac1 or Cdc42 GTPases were used as substrates in the presence of 100 µg of TM-TcdA or TM-TcdB (data not shown).

### Cytotoxicity of TcdA and TcdB mutants

To determine whether the mutations introduced into TcdA and TcdB were sufficient to reduce cytotoxicity, a quantitative bioluminescent cell viability assay using the IMR-90 fibroblast cell line was established. This assay was also employed to measure the ability of specific antibodies or chemical treatment to neutralize toxin-induced cytotoxicity. Both wt and mutant toxins were evaluated in the assay and the results are summarized in Table 1. Mutant toxins were orders of magnitude less cytotoxic than their corresponding wt toxins. Cytotoxic EC_50_ values for DM-TcdA and DM-TcdB increased by approximately 14 000-fold for DM-TcdA and 1600-fold for DM-TcdB. Introduction of the C698A cysteine protease mutation into DM-TcdB to generate TM-TcdB resulted in a fivefold further reduction in cytotoxicity (EC_50_ shift of 1600 to 8000-fold relative to wt TcdB). The corresponding C700A allele introduced into DM-TcdA (to create TM-TcdA) did not further reduce cytotoxicity. Interestingly, recent studies have shown that C698 protease mutations introduced in the context of wt TcdB do not independently prevent cytotoxicity but may influence the efficiency of Rho GTPase glucosylation (Chumbler *et al.*, 2012; Li *et al.*, 2013).

**Table 1.** Cytotoxicity of purified native and recombinant TcdA and TcdB toxins Recombinant plasmids were transferred to the *C. difficile* production strain and expressed toxins purified from 8 l fermentations (Methods). The cytotoxic potencies of the recombinant wt toxins were found to be indistinguishable from native toxins purified from VPI 10463 (List Biologicals).

Toxin	Genotype	IMR-90 EC_50_ (ng ml^−1^)	Fold reduction in cytotoxicity
Toxin A native	wtTcdA	0.9	1.0
Toxin A recombinant	wtTcdA	1.0	1.1
DM-TcdA	TcdA D285A/D287A	12 900	14 000
TM-TcdA	TcdA D285A/D287A/C700A	8600	9300
TM-TcdA formalin-treated	TcdA D285A/D287A/C700A	>100 000	>100 000
TM-TcdA antibody-treated	TcdA D285A/D287A/C700A	>100 000	>100 000
Toxin B native	wtTcdB	0.009	1.0
Toxin B recombinant	wtTcdB	0.006	0.7
DM-TcdB	TcdB D286A/D288A	14	1600
TM-TcdB	TcdB D286A/D288A/C698A	74	8200
TM-TcdB D270A/R273A/D461A/K463A	TcdB D286A/D288A/C698A/D270A/R273A/ D461A/K463A	127	14 000
TM-TcdB E970K/E976K	TcdB D286A/D288A/C698A/E970K/E976K	10 000	1 100 000
TM-TcdB formalin-treated	TcdB D286A/D288A/C698A	>100 000	>10 000 000
TM-TcdB antibody-treated	TcdB D286A/D288A/C698A	>100 000	>10 000 000

**Fig. 4.**
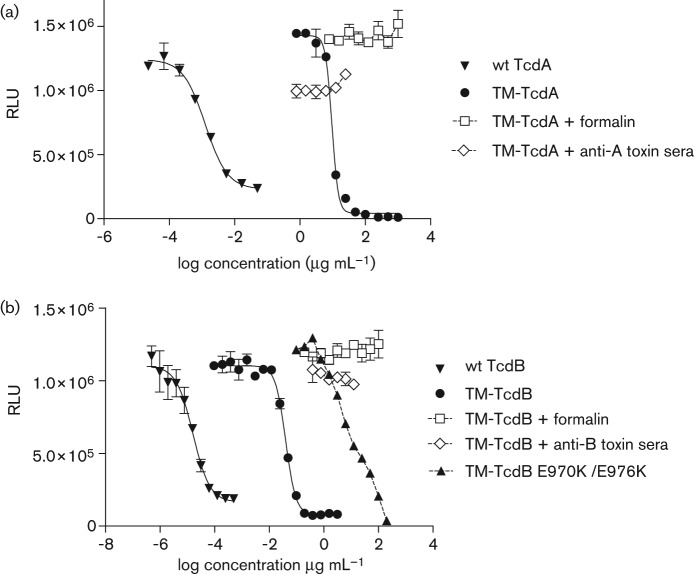
Neutralization of the cytotoxic activity of recombinant toxins with toxin-specific antibody or formalin pre-treatment. IMR-90 monolayers were incubated with serial dilutions of toxins and cell viability was determined after 3 days by measuring the release of intracellular ATP with a luciferase-based reagent. Error bars represent the standard deviations from results of duplicate serial dilutions. The relative cytotoxic activity of TM-TcdA (a) and TM-TcdB (b), with or without formalin or antibody pre-treatment, is illustrated. Neutralization of cytotoxic activity was achieved with a 1 : 10 dilution of toxin-specific rabbit polyclonal sera and by formalin pre-treatment.

Despite the ~10 000-fold reduction in cytotoxicity of the mutants relative to their wt toxins, both TM-TcdA and TM-TcdB retained the ability to kill IMR-90 cells in a concentration-dependent manner, with EC_50_ values of 8.6 µg ml^−1^ and 0.074 µg ml^−1^, respectively (Table 1, Fig. 4). This residual toxicity was blocked by pre-treatment of the TM toxins with toxin-specific antibodies or by treatment with a combination of formaldehyde and glycine. Pre-incubation with toxin-specific polyclonal sera (at 1 : 10 dilution) neutralized the cytotoxic activity of both TM toxins when tested at 25 µg ml^−1^. Likewise, formalin-treated TM-TcdA and TM-TcdB had no detectable effect on the viability of IMR-90 fibroblasts when tested at 1 mg ml^−1^ and 100 µg ml^−1^, respectively.

### Effect of mutant toxins on glucosylation of host-cell substrates

**Fig. 5.**
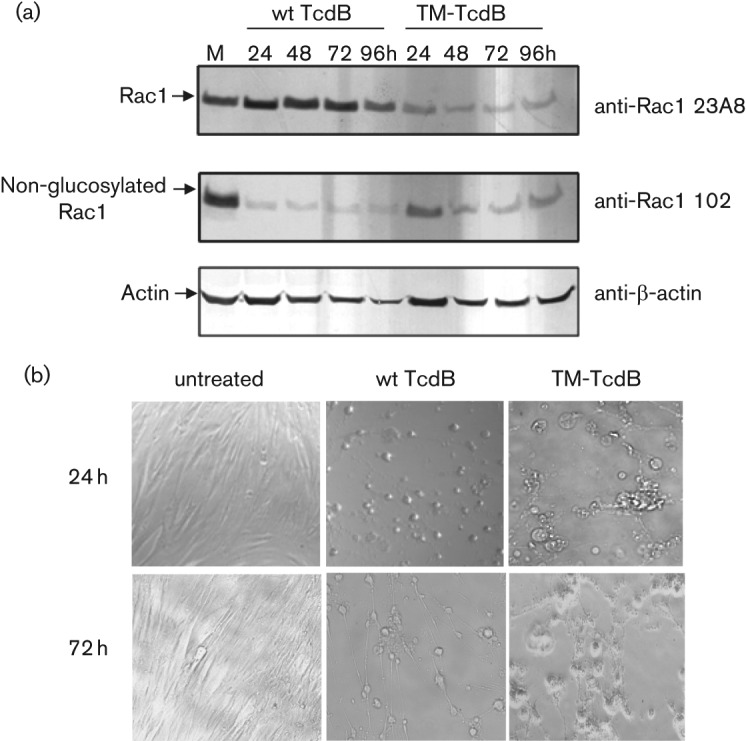
The toxicity of TM-TcdB is not associated with glucosylation of intracellular Rac1 GTPase and is phenotypically distinct from wt TcdB-induced cytotoxicity. (a) Lysates from IMR-90 cells exposed for different times to wt TcdB (0.5 ng ml^−1^) or TM-TcdB (100 ng ml^−1^) and mock-treated cells (M, 24 h) were separated by SDS-PAGE. Protein bands were transferred to nitrocellulose and probed with anti-Rac1 monoclonal antibodies which recognize either all forms of Rac1 protein (mAb 23A8) or only the non-glucosylated form of Rac1 (mAb 102). Actin levels were monitored as internal control. (b) Cellular morphology of cells at 24 h and 72 h time points. TM-TcdB induces cellular swelling rather than the rounding phenotype that is characteristic of wt TcdB toxin.

After demonstrating the lack of detectable GT activity of purified TM toxins, we evaluated the impact of cytotoxic levels of wt TcdB and TM-TcdB toxin on intracellular glucosylation of Rac1 GTPase in IMR-90 cells (Fig. 5). IMR-90 cells were harvested at different times following exposure to 0.5 ng ml^−1^ wt TcdB or 100 ng ml^−1^ TM-TcdB and the glucosylation status of Rac1 was determined by Western blot using a pair of monoclonal antibodies. The 23A8 mAb detects Rac1 independent of glucosyl modifications, while mAb 102 only binds to non-glucosylated Rac1 (Gerhard *et al.*, 2008). Following treatment of IMR-90 cells, the wt TcdB toxin induced glucosyl modification of intracellular Rac1 (i.e. loss of mAb 102 signal) and the cell-rounding phenotype characteristic of *C. difficile* toxins A and B (Fig. 5a, b). Under these conditions, the level of total intracellular Rac1 or actin remained relatively unchanged. Interestingly, cells treated with the TM-TcdB toxin showed consistently lower levels of Rac1 GTPase across a range of time points (24 h–96 h) compared with cells treated with wt TcdB toxin. Under these circumstances, non-glucosylated Rac1 GTPase was readily detected, in agreement with the lack of observed GT activity of the TM-TcdB toxin (Fig. 3 c, d). At the 24 h and 72 h time points, TM-TcdB treated cells appeared to be enlarged and morphologically distinct from wt TcdB treated IMR-90 cells (Fig. 5b).

**Fig. 6.**
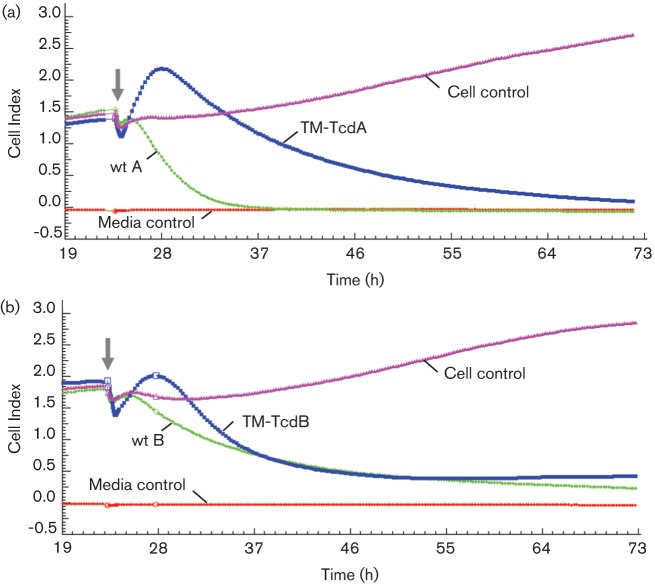
Real-time xCelligence impedence analysis of toxin-induced cytotoxicity. IMR-90 cells growing on a microelectrode matrix were incubated with native and recombinant toxins and cellular impedence monitored every 15 min for 3 days. Media control traces represent culture medium without IMR-90 cells, while cell control traces are recordings of cells without toxin treatment. (a) Treatment with 0.1 µg wt TcdA ml^−1^ or 250 µg TM-TcdA ml^−1^. (b) Treatment with 0.1 ng wt TcdB ml^−1^ or 100 ng TMTcdB ml^−1^. The solid arrows indicate the time when toxins were added to the cell monolayer.

For higher resolution kinetic analysis of cytotoxicity we used the xCelligence impedence-based system for continuous monitoring of IMR-90 cells in response to toxin and mutant toxin treatments. The electrical impedence measurement (or cell index) is proportional to the number and shape of viable cells growing on a microelectrode-coated surface (Ke *et al.*, 2011). In the experiment shown in Fig. 6, cells were incubated with cytotoxic concentrations of wt toxins or TM-TcdA and TM-TcdB mutants and measurements taken every 15 min for three days. Untreated control cells produced a steady increase in impedence that reflects growth of healthy fibroblasts. TM-TcdA- and TM-TcdB-treated cells induced a characteristic time-dependent response profile that was distinct from cells treated with wt toxins. IMR-90 cells treated with the wt toxins show a rapid decline in signal that reflects a loss of cellular contact through cell rounding and cell death. In contrast, cells treated with the TM toxins displayed a transient increase in impedence that accompanied a swelling of the cells observed by microscopic examination within the first 5 h of treatment. The subsequent decline in impedence correlated with a visible reduction in numbers of viable cells due to lysis of swollen cells. These results suggest that the mechanism of killing occurring at high concentrations of the TM toxins is distinct from the GT-dependent killing at much lower levels of the corresponding wt toxins.

### Evaluation of additional determinants of residual cytotoxicity

We evaluated other determinants potentially responsible for the residual cytotoxic activity of the TM genetic toxoids. Since the level of residual IMR-90 cytotoxicity was ~100-fold greater for TM-TcdB than for TM-TcdA (Table 1), these efforts focused on TM-TcdB. Structural modelling as well as structure–function studies of the TcdB toxin GT enzyme domain identified a number of specific amino acid residues responsible for the binding of UDP-glucose and GTPase protein substrates (Jank *et al.*, 2007b). Four of these mutations were introduced into TM-TcdB in an effort to abrogate substrate binding and to measure the impact on cytotoxicity. Alleles D270A and R273A eliminate two of the four side-chain contacts with the glucose moiety of UDP-glucose and alleles D461A and K463A prevent recognition of the GTPase substrates RhoA, Rac1, and Cdc42. The resulting mutant TcdB was found to be only marginally less toxic to IMR-90 cells than TM-TcdB (Table 1), a result which is consistent with the hypothesis that the residual cytotoxicity of both TM-TcdA and TM-TcdB antigens is independent of GT activity. Next we targeted a pair of conserved amino acids (E970 and E976) that are essential for pore formation, and which reside in a hydrophobic region (HR) within the translocation domain (Fig. 3a; von Eichel-Streiber *et al.*, 1992). Replacement of these residues with lysine eliminated pH-dependent pore-forming ability and significantly reduced the cytotoxicity of wt toxin B (Genisyuerek *et al.*, 2011). The same E970K/E976K substitution mutations introduced into TM-TcdB reduced residual toxicity by approximately 100-fold (Table 1, Fig. 4b). This result implicates poreformation as a mechanistic determinant of the residual cytotoxicity of TM-TcdB and related TM-TcdA toxins.

### Immunogenicity and efficacy of formalin-treated mutant toxins

**Fig. 7.**
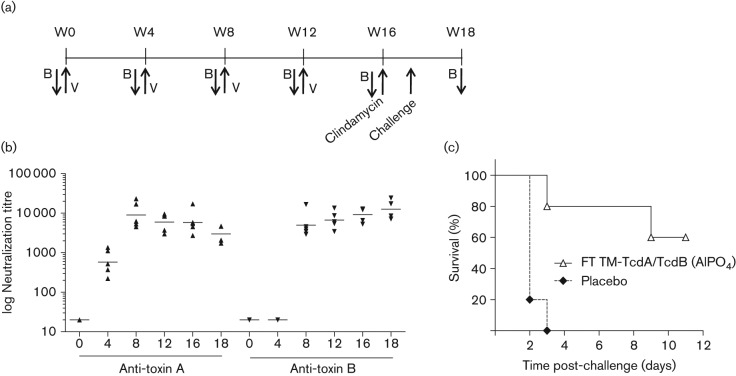
Immunogenicity and vaccine-induced protection in hamsters with formalin-treated TM-TcdA and TM-TcdB toxoids. (a) Groups of five Syrian golden hamsters were vaccinated (V) with 10 µg of each antigen in AlP0_4_ or saline placebo via intramuscular injection at weeks 0, 4, 8 and 12. Animals were bled for serum samples at indicated time points (B). Clindamycin was given orally at week 16 and animals challenged five days later with 5×10^3^ c.f.u. of *C. difficile* strain 630. (b) Anti-toxin A and B neutralization titres were determined at the indicated weeks (*x*-axis) using a toxin neutralization assay. (c) Hamsters were monitored for CDAD severity using a clinical scoring system. Animals with total severity scores ≥15 were euthanized. The study terminated at day 11 post-challenge.

Non-toxogenic formalin-treated TM-TcdA and TM-TcdB antigens were evaluated in hamsters to assess immunogenicity and protective efficacy (Fig. 7). Animals (*n* = 5) were vaccinated at four week intervals for four months with 10 µg of each antigen formulated with aluminium phosphate. A rapid increase of toxin A and toxin B neutralizing titres was observed, with anti-toxin A titres increasing at a faster rate than anti-toxin B titres, but with both reaching a maximum by the week 8 (post-dose 2) time point (Fig. 7b). Hamsters were treated with clindamycin at week 16 and challenged orally with *C. difficile* spores five days later. Within three days, all members of the group vaccinated with placebo developed severe CDAD symptoms and were euthanized (Fig. 7c). In contrast, 60 % of hamsters vaccinated with TM-TcdA and TM-TcdB toxoids survived until the study end point 11 days following *C. difficile* challenge.

## Discussion

Several immunotherapeutic approaches are being pursued for the control of CDAD. Early proof of concept that antibodies protect against CDAD has been demonstrated using monoclonal antibodies that neutralize TcdA and TcdB exotoxins (Lowy *et al.*, 2010). This immunotherapy is currently under evaluation in a Phase 3 trial (http://clinicaltrials.gov/show/NCT01241552). Toxoid vaccines are in Phase 1 (http://clinicaltrials.gov/show/NCT01706367) and Phase 2 (http://clinicaltrials.gov/show/NCT01230957). Manufacturing challenges associated with the production of a conventional toxoid vaccine include safe handling of the toxins, decontamination of heat-resistant spores formed as a natural by-product, adjusting the ratio of the two toxins made by the same production strain and low yield. The strategy described in this manuscript avoids these issues and is the basis for the approach taken for one of the investigational vaccines (http://clinicaltrials.gov/show/NCT01706367). Newly available genetic tools were utilized to develop a novel platform for the independent overexpression of full-length genetically inactivated toxins in a non-toxigenic, non-sporogenic *C. difficile* strain. The recombinant technology described herein provides a major advancement for the production of *C. difficile* toxoid-based vaccines being developed to relieve the high burden attributed to CDAD in healthcare facilities. The utility of this approach was demonstrated in preliminary studies in which recombinant toxoids generated robust anti-toxin neutralizing antisera and protected hamsters from development of severe CDAD.

Using a *C. difficile* plasmid replicon and a constitutive *C. sporogenes fdx* gene promoter to drive recombinant antigen expression, we produced TcdA or TcdB toxins in small-scale cultures at levels that were substantially in excess of yields previously optimized through media studies for a vaccine production strain (Fang *et al.*, 2009). Purified recombinant TM-TcdA and TM-TcdB toxins engineered to eliminate GT and autoproteolytic processing activities showed a ~10 000-fold reduction in cytoxicity. However, the detection of some residual toxicity, in the *μ*g ml^−1^ range for TM-TcdA and sub-*μ*g ml^−1^ range for TM-TcdB, prompted us to investigate the underlying mechanism to identify additional measures to assure vaccine antigen safety.

Multiple observations support the conclusion that the cytotoxicity that remains associated with TM-TcdA and TM-TcdB antigens is unrelated to GT activity. These include, (i) the complete lack of GT activity detectable in purified TM toxin preparations, (ii) the inability to detect glucosylated GTPase substrates in fibroblasts treated with cytotoxic levels of TM-TcdB, (iii) the absence of the cell-rounding phenotype that is characteristic of GT-associated toxicity, and (iv) the minimal impact on cytotoxicity conferred by additional amino acid substitutions that eliminate the ability of the TcdB GT to recognize UDP-glucose and GTPase substrates.

We demonstrated that by introducing the E970K/E976K mutations, most of the residual cytotoxicity associated with TM-TcdB could be eliminated. This pair of glutamate residues has been previously shown to play a role in pore formation. Unlike the TcdB native toxin that causes leakage of cell membranes, the TcdB E970K/E976K mutant was shown to be completely incapable of releasing of rubidium (^96^Rb^+^) from preloaded CHO cell monolayers (Genisyuerek *et al.*, 2011). Similarly, in a trans-epithelial electrical resistance assay, the E970K/E976K mutant toxin largely reduced the permeability of epithelial cell monolayers observed with wt toxin B (Genisyuerek *et al.*, 2011). The morphological changes that accompany treatment of IMR-90 cells with the TM-TcdA and TM-TcdB toxins described in this report are consistent with a pore-induced membrane leakage mechanism characterized by the slow enlargement of cells and subsequent cell death. Enhanced membrane permeability may also explain the lower levels of cytosolic Rac1 GTPase detected in cells treated with TM-TcdB relative to cells treated with wt TcdB (Fig. 5a).

It has been proposed that poreformation induced by clostridial glucosylating toxins is linked to toxin translocation (Barth *et al.*, 2001; Giesemann *et al.*, 2006). The functional significance of TcdB glutamates 970 and 976 was first inferred from the conservation of these acidic residues across related clostridial toxins within a HR required for vesicular membrane translocation and GT delivery (Genisyuerek *et al.*, 2011). Since a reduction in toxicity was only observed when these glutamates were substituted by lysine, it is believed that they play a pivotal role in pore formation and the pH-dependent insertion of the toxins into vesicular membranes. Although we did not introduce analogous substitutions in the orthologous aspartate 972 and aspartate 978 residues of the TM-TcdA toxin and demonstrate mitigation of toxicity, we predict that these residues are also likely to be determinants contributing to the residual cytotoxicity of this mutant toxin.

Chemical treatments such as formalin have traditionally been used to detoxify toxin-based vaccines and this is the approach being used by one of the investigational vaccines (http://clinicaltrials.gov/show/NCT01230957). Chemical treatment may negatively impact antigenicity and/or immunogenicity and complicate manufacturing. We therefore investigated whether genetically modified antigens could be produced that avoid the need for chemical treatment steps. During our characterization of recombinant *C. difficile* TcdA and TcdB, we were able to differentiate two discrete mechanisms of toxicity that can be eliminated by targeted mutation. The primary one, associated with the well-established N-terminal GT activity of the toxins, is responsible for toxicity in the sub-ng ml^−1^ range. However, we also confirm the existence of an independent secondary mechanism based on membrane leakage induced by pore formation. This was detected in the low-*μ*g ml^−1^ range. We observed that genetic neutralization of both mechanisms in TcdB does not completely eliminate toxicity, as residual cytotoxicity at concentrations of >10 µg ml^−1^ was still detected (Table 1). We speculate that the source of this remaining toxicity may be related to incomplete suppression of pore-forming activity by the double E970K/E976K mutation, or due to saturation of potentially multifunctional host-cell receptors mediating endocytosis of the TcdB protein (Zemljic *et al.*, 2010). Nevertheless, we demonstrated that complete neutralization could be achieved when the TM mutant toxins were pre-incubated with specific antibodies. We also demonstrated that complete removal of the residual cytotoxicity of TM mutant toxins was readily achieved by formalin inactivation. We will show separately that chemical neutralization of the residual cytotoxicity of TM mutant toxins can be accomplished without impairing the ability of the vaccine to generate neutralizing antibodies (Justin Moran, unpublished results).

In summary, we describe a strategy for the production of recombinant *C. difficile* toxin antigens, and highlight distinct cytotoxic mechanisms that need to be addressed prior to the advancement of vaccine candidates. Ultimately, identifying an appropriate process for the manufacturing of a safe toxoid-based *C. difficile* vaccine will likely require striking a balance between genetic modification and chemical treatment strategies.
